# Transcriptome-based profiling of programmed cell death signatures identifies two osteoarthritis subtypes and the hub gene DNM1L

**DOI:** 10.3389/fimmu.2026.1816005

**Published:** 2026-08-27

**Authors:** Xiaoning Lin, Yujian Lan, Xuan Lin, Yue Lai, Jianlin Shen, Huan Liu

**Affiliations:** 1 Department of Orthopedics, Affiliated Hospital of Putian University, Putian, China; 2 School of Integrated Traditional Chinese and Western Medicine, Southwest Medical University, Luzhou, China; 3 Department of Orthopaedics, The Affiliated Traditional Chinese Medicine Hospital, Southwest Medical University, Luzhou, China; 4 Department of Environmental and Biological Engineering, Putian University, Putian, China; 5 Intervention Department, Longyan Second Hospital, Longyan, China; 6 Central Laboratory, Affiliated Hospital of Putian University, Putian, China

**Keywords:** biomarkers, integrative bioinformatics, machine learning, osteoarthritis, programmed cell death

## Abstract

**Background:**

Osteoarthritis (OA) poses a significant global health risk, with programmed cell death (PCD) playing a key role in its development.

**Methods:**

This study utilized integrated bioinformatics tools to discover new biomarkers and therapeutic targets for OA. A comprehensive set of 1,254 PCD-related genes encompassing 12 programmed cell death modalities was identified through literature review and pathway analysis. Gene expression data for OA patients and healthy controls were obtained from the GEO database, leading to the classification of patients into two subtypes, Cluster 1 and Cluster 2, based on ssGSEA scores. Immune infiltration analysis and LASSO regression were employed to construct classifiers for these subtypes, followed by validation with external datasets. SVM and RF machine learning techniques were used to identify hub genes. Western blotting and qRT-PCR were performed to validate hub gene expression in OA synovial tissues.

**Results:**

The analysis revealed two OA subtypes with distinct biological processes. A robust classifier was developed, and DNM1L emerged as the sole hub gene linked to PCD in OA. The LASSO classifier achieved an AUC of 1.000 in the training cohort but showed reduced performance in external validation cohorts, indicating the need for further validation. Both immunoblot and qRT-PCR confirmed increased DNM1L expression in OA patients.

**Conclusion:**

This study successfully classified OA into two subtypes and identified DNM1L as a PCD-associated candidate gene that may contribute to OA pathogenesis.

## 1 Introduction

Osteoarthritis (OA) represents the most prevalent form of degenerative joint disease among the elderly, significantly diminishing quality of life and contributing to disability. The incidence of OA is on the rise, partly attributable to an increase in risk factors such as obesity, sedentary lifestyles, and previous joint injuries (1). This condition can affect a range of synovial joints, with the knee being the most commonly involved, followed by the lumbar spine, cervical spine, hands, ankles, and hips (2). Although the pathological mechanisms underlying OA can vary among different joint types, they typically manifest as a gradual deterioration of articular cartilage, accompanied by recurrent instances of synovitis and bone remodeling (3). With the global population expanding and life expectancy increasing, the management of OA has become a pressing public health concern due to its widespread occurrence and associated disabilities. Despite extensive research aimed at unraveling the complexities of osteoarthritis, the precise mechanisms and associated risk factors remain poorly understood. Early detection is crucial for enhancing the diagnosis and management of OA; however, it poses a significant challenge. Radiographic imaging and clinical evaluations are the primary diagnostic tools currently in use (4). Nevertheless, OA is frequently diagnosed only after the manifestation of clinical symptoms, which typically occurs when structural damage has already become significant (5). Moreover, differentiating OA from other arthritic conditions can be problematic due to overlapping clinical symptoms and similar pathological characteristics (6). Common features present in arthritic diseases include inflammation, deterioration of connective tissue, pain, and stiffness (7). These diagnostic challenges not only lead to substantial confusion but also result in delayed implementation of effective treatment strategies. In conclusion, there is still an absence of dependable biomarkers with high sensitivity and specificity necessary for the early and accurate diagnosis of OA, thereby underscoring the need for identifying specific biomarkers. Conventional OA classification systems, such as the Kellgren–Lawrence radiographic grading, rely primarily on macroscopic structural alterations including joint space narrowing and osteophyte formation (8, 9). Increasing evidence indicates that OA represents a heterogeneous disease with distinct molecular phenotypes that may differ in inflammatory status, metabolic activity, and treatment response (10, 11). Therefore, transcriptome-based molecular stratification combined with machine learning approaches may provide additional insights beyond traditional structural classification and facilitate the identification of clinically relevant OA subtypes and potential therapeutic targets (12, 13).

Cell death is categorized based on its underlying mechanisms into accidental cell death (ACD) and programmed cell death (PCD). PCD is a mode of active cell death (14) regulated by various genes, mainly Apoptosis, Autophagy, Cuproptosis, Entotic cell death, Ferroptosis, Lysosome-dependent cell death, Necroptosis, Netotic cell death, Oxeiptosis, Parthanatos, Alkaliptosis and Pyroptosis (15). Among these, Apoptosis is a specific type of PCD that facilitates the removal of unnecessary or damaged cells in a regulated manner. This process encompasses coagulation, nuclear fragmentation, and nucleolysis. The resulting apoptotic bodies are then engulfed by macrophages, leaving adjacent cells unaffected, thereby preventing an inflammatory response (16). Autophagy-dependent cell death involves a complex, multi-step lysosomal degradation process that aids in metabolic adaptation and nutrient recycling (17). Recently, copper toxicity has been recognized as a novel form of cell death, arising from copper metabolism via the lipidated TCA cycle, and is closely linked to serious health conditions (18). The death of neutrophils occurs through the release of neutrophil extracellular traps (NETs), which are filamentous structures released during infections or injuries (19). Ferroptosis, another type of cell death, is characterized by the accumulation of lipid hydroperoxides to toxic levels, and it is dependent on iron (20). Lysosome-dependent cell death is governed by hydrolytic enzymes and occurs following the permeabilization of membranes, leading to enzyme release into the cytoplasm (21). Historically, necrosis has been viewed as an unregulated form of cell death; however, recent findings indicate that it can be initiated and maintained, resulting in a form of necroptosis associated with necrotic body morphogenesis (22). Entotic cell death refers to an active invasion occurring solely within the interior and adjacent regions of intact cells, representing a non-apoptotic pathway that does not involve the activation of apoptotic executioners (23). Oxeiptosis, characterized as a non-inflammatory and caspase-independent form of PCD, is instigated by reactive oxygen species (ROS) and can activate other forms of PCD, including pyroptosis, apoptosis, ferroptosis, necroptosis, and autophagy-dependent cell death (24). Parthanatos results from hyperactivation of the PARP-1 nuclease, while alkaliptosis is a newly identified type of programmed cell death (PCD) that is heavily dependent on intracellular alkalinization processes (25, 26). Pyroptosis is an inflammatory variant of PCD, typically marked by the formation of membrane pores that compromise cellular integrity and ultimately cause cell lysis (27). Research into PCD has significantly advanced the development of diverse therapeutic agents. For instance, BCL-2 inhibitors have emerged as a treatment option for tumors such as triple-negative breast cancer by influencing the apoptosis pathway (28). Furthermore, the induction of mediated focal death represents a promising and innovative strategy in the field of anti-tumor therapies (29).

DNM1L (Drp1), a key regulator of mitochondrial fission, is upregulated in human and mouse OA cartilage, where it drives excessive mitochondrial fragmentation, cytochrome c release, and chondrocyte apoptosis through ERK1/2-mediated phosphorylation. This DNM1L-mediated fission also amplifies mitochondrial ROS accumulation, further exacerbating cartilage degradation (30).

A recent study has shown that programmed cell death is strongly associated with OA. Programmed cell death may be an important risk factor for osteoarthritis. In general, different types of programmed cell death play different roles in OA. Apoptosis contributes to the loss of chondrocytes and degradation of the extracellular matrix (31), while pyroptosis aggravates cartilage injury by triggering inflammatory responses (32). Necroptosis promotes inflammatory propagation and tissue damage (14), whereas ferroptosis induces chondrocyte injury through iron-dependent lipid peroxidation (33). Then autophagy can inhibit the deterioration of OA, thus protecting cartilage. Cell death pathways do not function in isolation; rather, they exhibit extensive crosstalk. Oxidative stress, mitochondrial dysfunction, and inflammatory signaling serve as shared upstream drivers that can engage apoptosis, ferroptosis, pyroptosis, and other PCD (34). Therefore, focusing on a single PCD modality may only capture a limited aspect of OA pathogenesis and fail to represent the complex cellular fate landscape. An integrated transcriptomic analysis incorporating multiple PCD-related genes may provide a more comprehensive understanding of OA molecular heterogeneity and facilitate the identification of novel disease-associated signatures.

## 2 Data and methods

### 2.1 PCD-related gene collection

PCD-related genes contain 12 key regulatory genes for PCD patterns as described below. The genes were collected from the GSEA gene collection, KEGG, review articles and manual curation. The final gene list is the tandem regulated genes for the 12 PCD modes. A total of 7 Alkaliptosis genes, 580 Apoptosis genes, 367 Autophagy genes, 14 Cuproptosis genes, 15 Entotic cell death genes, 88 Ferroptosis genes, 220 Lysosome-dependent cell death genes, 101 Necroptosis genes, 8 Netotic cell death genes, 5 Oxeiptosis genes, 9 Parthanatos genes, and 52 Pyroptosis genes. Finally, 1254 PCD-related tandem genes were included in the analysis (**Supplementary Table 1**).

### 2.2 Gene expression profiling data

The GSE236924 gene expression profiling dataset was downloaded from the GEO database (https://www.ncbi.nlm.nih.gov/geo/) and used as the primary discovery cohort for all analyses. A total of 96 samples (89 OA samples and 7 control samples) were included in the GSE236924 data series. Gene expression was detected using GPL570 [HG-U133_Plus_2] Affymetrix Human Genome U133 Plus 2.0 Array.

### 2.3 ssGSEA analysis

Based on the expression of each gene in PCD and the corresponding gene sets, the OA samples in the GSE236924 dataset were scored by enrichment analysis using the ssGSEA method in the GSVA (35) package.

### 2.4 Unsupervised clustering

PCD-specific differentially expressed genes play a unique role in OA. Therefore, to further explore this point, we performed unsupervised clustering of all OA patients using the “ConsensusCluster” package in R 4.3.2, and categorized the patients into subtypes with different clinical characteristics and further distinguished them using the ssGSEA score of PCD as a reference. The classified patients were then observed, and the discriminative ability of the subtypes was evaluated by principal component analysis, and the differences in the ssGSEA scores of the 12 PCDs between subtypes were calculated by wilcox.test.

### 2.5 Differential expression analysis between subtypes

Using the “limma” package in R 4.3.2 software (36), different subtypes of the GSE236924 matrix data were normalized, log2 transformed and screened for DEGs. |log2FC| > 0.585, p-value < 0.05 were considered as the threshold level of statistical significance for DEGs samples. The DEGs identified between the two PCD-related OA subtypes were retained for subsequent analyses, including LASSO-based subtype classification.

### 2.6 Functional enrichment analysis of DEGs

Significant DEGs were further analyzed for Gene Ontology (GO) functional and Kyoto Encyclopedia of Genes and Genomes (KEGG) pathway enrichment analysis using the “Bioconductor” and “GOplot” software packages in R 4.3.2. An adjusted p-value of <0.05 was used as the cut-off criterion.

### 2.7 Analysis of immune infiltration in patients with different subtypes of the disease

CIBERSORT has been widely validated for immune deconvolution of bulk transcriptomes from complex tissues, including OA cartilage and synovium, and the LM22 signature has been successfully applied in multiple OA immune-landscape studies (37, 38). In order to investigate whether there were differences in immune cell infiltration in different disease subtypes, we performed cibersort (39) immune infiltration analysis based on clustered grouping of OA samples and the expression of each corresponding gene, and then calculated the differences between groups based on the expression of immune checkpoint genes using wilcox.test. Subsequently, the immune correlation score was calculated for each sample based on the expression of each gene corresponding to each sample using estimate (40).

### 2.8 Drug sensitivity analysis

In order to explore the guiding significance of disease typing results for arthritis chemotherapy treatment, drug sensitivity analysis was performed here based on the expression of each OA sample and the GDSC2 database using the R package oncoPredict. The samples were then grouped based on sample typing, and the differences in specific drug sensitivity between groups were counted (t.test).

### 2.9 Disease typing classifier training and validation

In order to expand the potential application value of disease typing as described above, classifiers that can be used to distinguish between different disease subtypes were developed in this study. PCD-related genes were selected from the differential genes of GSE236924 typing. The R package glmnet was used to construct a LASSO regression model (binomial), and the cv.glmnet function was applied to perform cross-validation for selecting the optimal penalty parameter (λ). The model was trained to obtain a classifier that can typify the samples (the data of OA were divided into two groups of train and test according to 1:1), and the classification results of each group were predicted using the obtained model. The corresponding probability value of each sample was calculated, and the pROC package was used to compare the predicted probabilities with the real classification for ROC analysis and calculate the AUC value.

Based on the above classifiers, outside validation was performed using the external dataset GSE55235. Based on the expression data of GSE55235 and the set of 12 PCD genes, ssGSEA analysis was first performed. Then clustering analysis was performed based on ssGSEA scores. Finally, external data validation was performed based on the classification model obtained above.

### 2.10 Core gene screening related to PCD

To investigate differences in PCD activity between OA and control samples in the GSE236924 dataset, ssGSEA was performed using the GSVA package based on the 12 curated PCD-related gene sets. The ssGSEA scores of each PCD modality were compared between the OA and control groups, and PCD modalities showing significant differences between the two groups were retained for subsequent analysis.

For each PCD modality showing a significant difference between OA and control samples, the samples were further stratified into high- and low-activity groups according to the median ssGSEA score of the corresponding PCD modality. Differentially expressed genes between the high- and low-activity groups were identified using the criteria of |log2FC| > 0.585 and p-value < 0.05.

The differentially expressed gene sets obtained from the individual PCD modalities were then compared using the UpSetR package to identify genes shared across multiple PCD modalities. The overlapping genes were considered candidate genes associated with multiple forms of PCD and were subsequently subjected to SVM-RFE and Random Forest analyses for further feature selection. The genes identified by both machine-learning approaches were considered candidate hub genes.

### 2.11 External validation of the identified hub genes

The GSE29746 and GSE82107 datasets were used as independent external validation cohorts. ROC curve analysis was conducted using the pROC package in R to validate the diagnostic value of the identified hub genes in independent osteoarthritis cohorts. The area under the ROC curve (AUC) was calculated to quantify the diagnostic performance of each hub gene, with AUC values ranging from 0.5 to 1.0. Higher AUC values indicate greater discriminatory ability.

### 2.12 Experimental validation

Synovial tissue samples were collected from the knees of six patients undergoing arthroscopic surgery: three elderly patients with osteoarthritis and three young patients. Informed consent was obtained from all participants, and the study was approved by the Ethics Committee of Putian University Affiliated Hospital (Putian, China). The research adhered to the principles outlined in the Declaration of Helsinki.

Total RNA was extracted from synovial tissue samples of the control and osteoarthritis groups using the SPINeasy DNA/RNA/Protein All-in-One Kit (MP Biomedicines, California, USA). RNA concentration was measured with an ultra-micro spectrophotometer (Impen, Munich, Germany), and reverse transcription was performed using PrimeScript RT Master Mix (Takara, Dalian, China). Quantitative real-time PCR (qRT-PCR) was employed to analyze the mRNA expression of dynamin 1-like (DNM1L). The DNM1L primer sequences were: forward primer, 5’-GCTCAGAGGTCTTCAACA-3’; reverse primer, 5’-TCTCTCTTCCACCCATTCT-3’. qRT-PCR was conducted using Hieff qPCR SYBR Green Master Mix (High Rox Plus, Yeasen, Shanghai, China) on an ABI StepOne Plus detection system (Thermo Fisher Scientific, Massachusetts, USA). Amplification conditions were as follows: 95 °C for 5 minutes, followed by 40 cycles of 95 °C for 10 seconds, 60 °C for 20 seconds, and 70 °C for 20 seconds. All primer pairs achieved amplification efficiencies of approximately 100%. Intra-assay precision was evaluated by performing triplicate technical replicates for each sample on the same plate. Dissociation curve analysis was performed to confirm primer specificity, and β-actin served as an internal control. Calculated the expression level of target RNA based on the threshold cycle (Ct), R = 2 ^-ΔΔCT^.

Total protein was extracted from synovial tissue samples of the control and osteoarthritis groups using the SPINeasy DNA/RNA/Protein All-in-One Kit (MP Biomedicines, California, USA). Protein concentration was determined using the BCA protein assay kit (Beyotime, Shanghai, China). Protein samples (40 µg/lane) were separated by 10% sodium dodecyl sulfate-polyacrylamide gel electrophoresis (SDS-PAGE) and transferred to a membrane. The membrane was incubated with an anti-DNM1L antibody (1:1000; ABclonal, Wuhan, China; catalog #A2586) at 4 °C, followed by a goat anti-rabbit horseradish peroxidase (HRP)-conjugated secondary antibody (1:3000; Proteintech Group, Chicago, USA) at room temperature for 1 hour. Protein bands were visualized using an enhanced chemiluminescence (ECL) system (Beyotime, Shanghai, China) and imaged with an Invitrogen gel imager (iBright FL1000, Thermo Fisher Scientific, Waltham, MA, USA). Band intensities were quantified using ImageJ software (NIH, Bethesda, MD, USA). Data are presented as mean ± SD. Distribution normality was assessed by the Shapiro–Wilk test. For comparisons between OA and young control groups, unpaired two-tailed Student’s t-test was used for normally distributed data; otherwise, the Mann–Whitney U test was applied. P < 0.05 was considered statistically significant. All statistical analyses were performed using GraphPad Prism 9.5.1.

## 3 Results

### 3.1 PCD scoring

**Figure 1:**
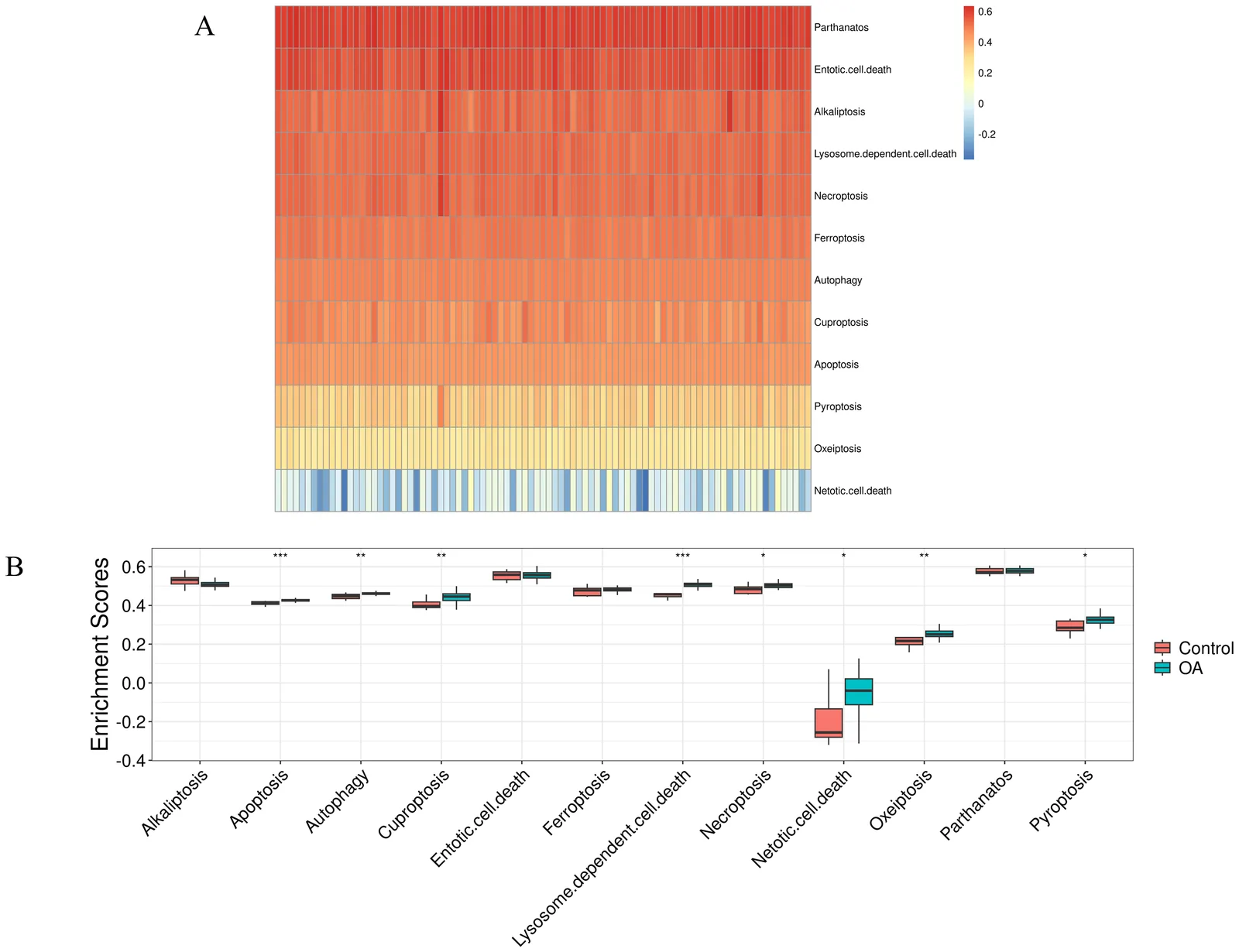
Scoring of OA samples in the GSE236924 dataset using the ssGSEA method based on the set of 12 programmed cell death (PCD) genes. **(A)** Heatmap of ssGSEA scoring results. **(B)** Boxplot demonstrating between-group differences in PCD scores for OA and control groups. *P < 0.05; **P < 0.01; ***P < 0.001.

Scoring the OA samples according to the set of genes for 12 PCDs, we found that the main focus was on 4 cell death modes such as Apoptosis, Pyroptosis, Oxeiptosis and Netotic cell death (**Figure 1A**). Box line plots (**Figure 1B**) then demonstrated intergroup differences in PCD scores between OA and control groups for Apoptosis, Autophagy, Cuproptosis, Lysosome-dependent cell death, Necroptosis, Netotic cell death, Oxeiptosis and Pyroptosis.

### 3.2 Identification of subtypes

**Figure 2:**
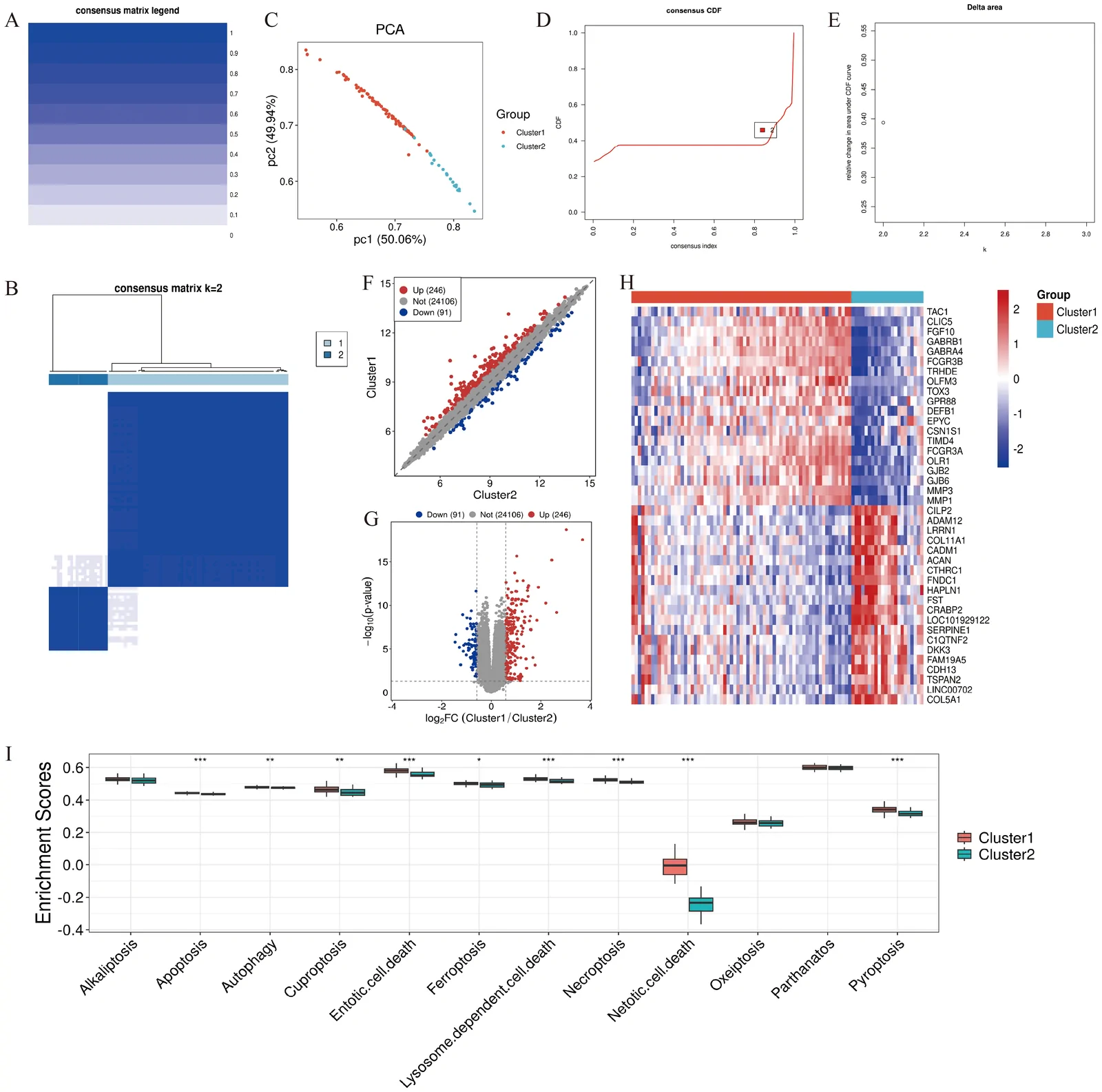
Identification of OA disease subtypes **(A–C)** Screening of molecular subgroups by unsupervised learning cluster analysis. **(D)** Consensus Cumulative Distribution Function (CDF) plot. **(E)** Delta area plot for determining the optimal cluster number. **(F, G)** Differential expression scatter plot between Cluster1 and Cluster2. **(H)** Heatmap of differential expression between Cluster1 and Cluster2. **(I)** Comparison of the expression of 24 PCD-related degs between Cluster1 and Cluster2. (***Means P < 0.001; **Means P < 0.01; *Means P < 0.05.).

Unsupervised cluster analysis revealed two disease subtypes, which we named Cluster1 and Cluster2 (**Figures 2A, B**). We then performed a series of analyses to distinguish the differences between these two subtypes. PCA analysis revealed that Cluster1 and Cluster2 were better differentiated (**Figure 2C**). By comparing the differential gene expression between their two subtypes, we found that there were significant differences between the Cluster1 and Cluster2 subtypes, and the differences were statistically significant (**Figures 2D–F**). Next, we compared the PCD differences between the two subtypes and found that the two subtypes, Cluster1 and Cluster2, showed significant differences in Entotic cell death, Lysosome-dependent cell death, Necroptosis, Ferroptosis, Autophagy, Cuproptosis, Apoptosis, and Pyroptosis. There were significant differences between the 9 PCDs (**Figure 2G**). This is very similar to the results of our previous analysis of PCD ssGSEA scores in OA patients and healthy individuals.

### 3.3 Functional enrichment analysis of DEGs between different subtypes

**Figure 3:**
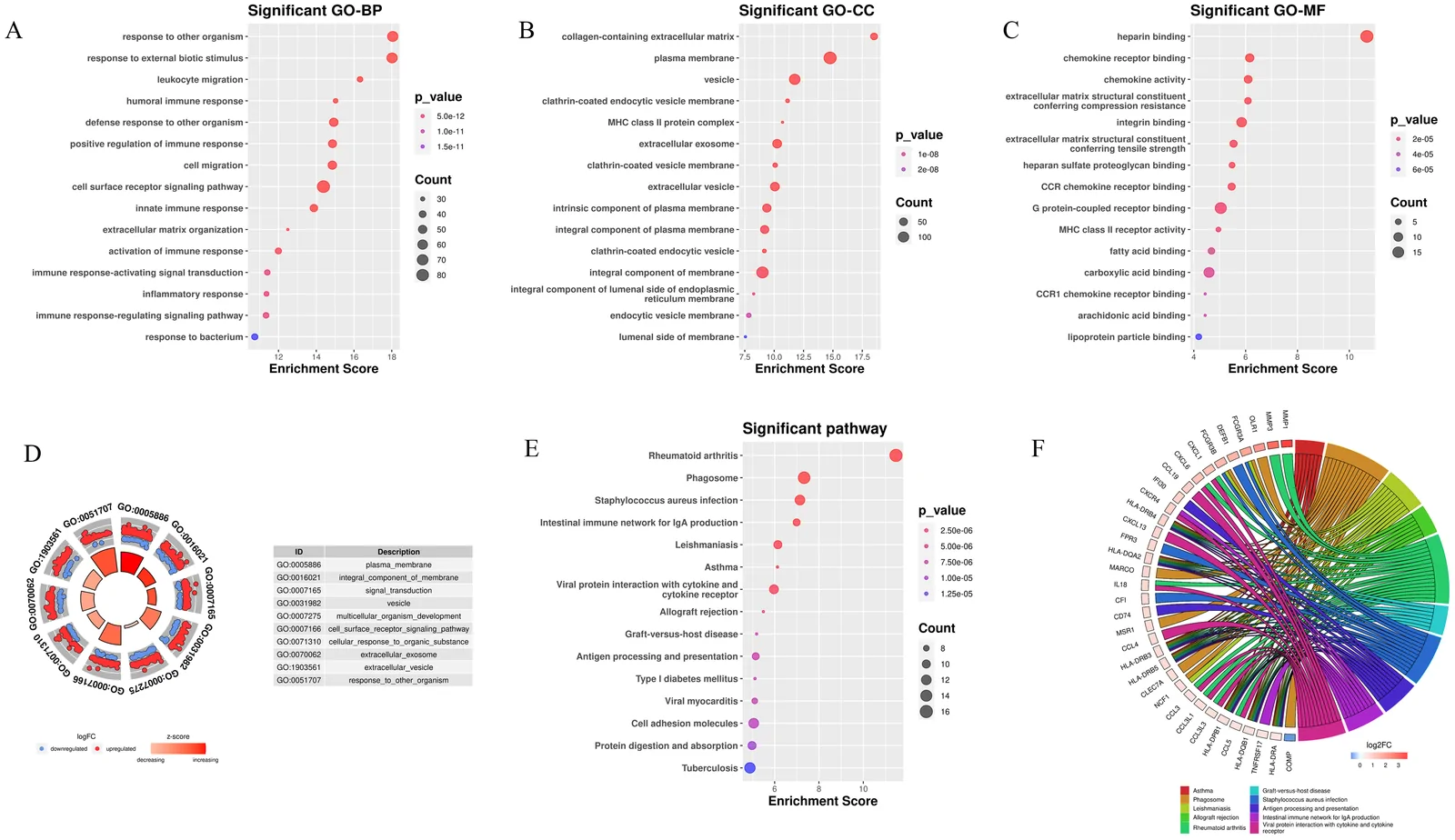
GO and KEGG functional enrichment of DEGs between different subtypes. **(A–D)** Results of GO (BP,CC,MF) enrichment of DEGs between different subtypes, respectively. **(E, F)** KEGG enrichment results of DEGs between different subtypes.

Functional enrichment analysis of the two different subtypes of DEGs showed that their Biological Process (BP) was mainly focused on the response to other organisms, response to external biological stimuli, leukocyte migration, humoral immune response, and defense response to other organs. Cellular Component (CC) mainly focuses on collagen-containing extracellular matrix, plasma membrane, vesicle, clathrin-coated endocytic vesicle. Molecular Function (MF) was mainly focused on coagulation factor binding, coagulation factor receptor binding, coagulation factor activity, structural components of the extracellular matrix, provision of antistress properties, and integrin binding (**Figures 3A–D**). KEGG enrichment in Viral myocarditis, Cell adhesion molecules, Protein digestion and absorption, Tuberculosis was strongly associated with programmed cell death (**Figures 3E, F**).

### 3.4 Analysis of immune infiltration between different subtypes

**Figure 4:**
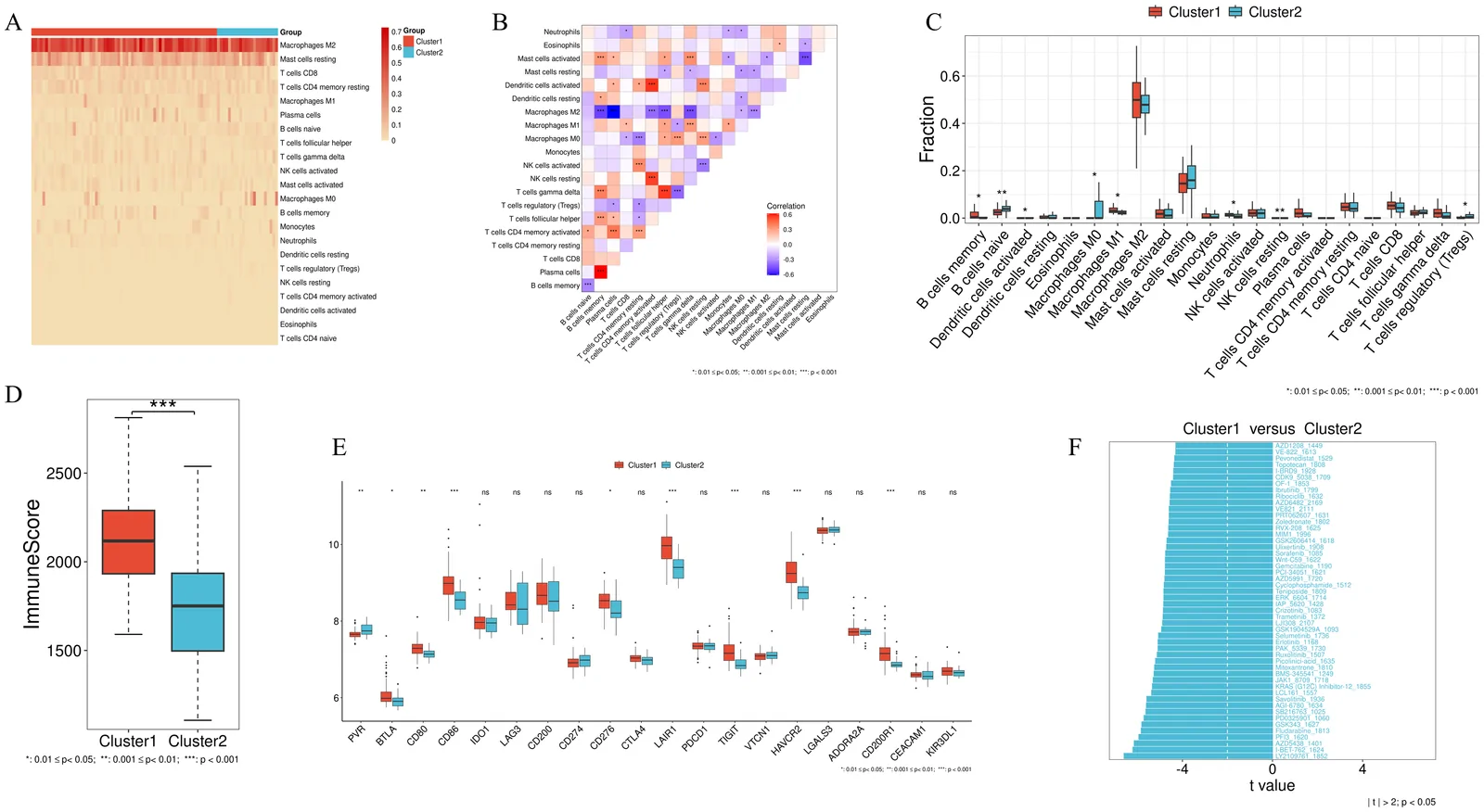
Levels of immune cell infiltration in two OA subtypes. **(A)** Comparison of immune cell infiltration levels of two OA subtypes. **(B)** Heat map of immune cell correlation of two OA subtypes. **(C)** Box line plot of the results of differences in immune cell infiltration between different typing groups. **(D)** Histogram of results of differences in immune scoring between different typing groups. **(E)** Box line plot of immune checkpoints between different typing groups. **(F)** Bar graph of IC50 differences between different fractions (only the top 50 drugs with absolute t values are shown here). *P < 0.05; **P < 0.01; ***P < 0.001.

Based on the gene expression data of Cluster1 and Cluster2 subtypes in the dataset, we analyzed the specific immune changes associated with the development of OA (**Figures 4A–C**). The results showed that significant differences between Cluster 1 and Cluster 2 included B cells naive, resting NK cells, activated dendritic cells, memory B cells, M1 macrophages, M0 macrophages, neutrophils, and regulatory T cells (Tregs). Correlation analysis revealed strong associations between specific immune cell subsets, such as plasma cells and memory B cells, gamma delta T cells and follicular helper T cells, resting NK cells and activated CD4 memory T cells, and activated dendritic cells and activated CD4 memory T cells. Subsequently, we performed immunoscoring based on the expression of each sample, and the results showed that the immunoscore of Cluster1 was significantly higher than that of Cluster2 (**Figure 4D**). We performed an analysis of differences in immune checkpoints for the disease subtypes, and the results showed that there were significant differences between the two subtypes in HAVCR2, LAIR1, CD200R1, TIGIT, CD86, CD80, PVR, BTLA, and CD276. A total of nine immune checkpoints were identified (**Figure 4E**).

### 3.5 IC 50 analysis

In order to investigate the significance of disease typing results for the guidance of OA chemotherapy treatment, IC50 drug analysis was performed, and the results showed that there were significant differences between the two subtypes of LY2109761_1852, I-BET-762_1624, Fludarabine_1813, and PFI3_1620, as shown in the graph demonstrating the top 50 drugs (**Figure 4F**), which may be potential drugs for the treatment of different subtypes of OA.

### 3.6 Disease typing classifier training and external validation

**Figure 5:**
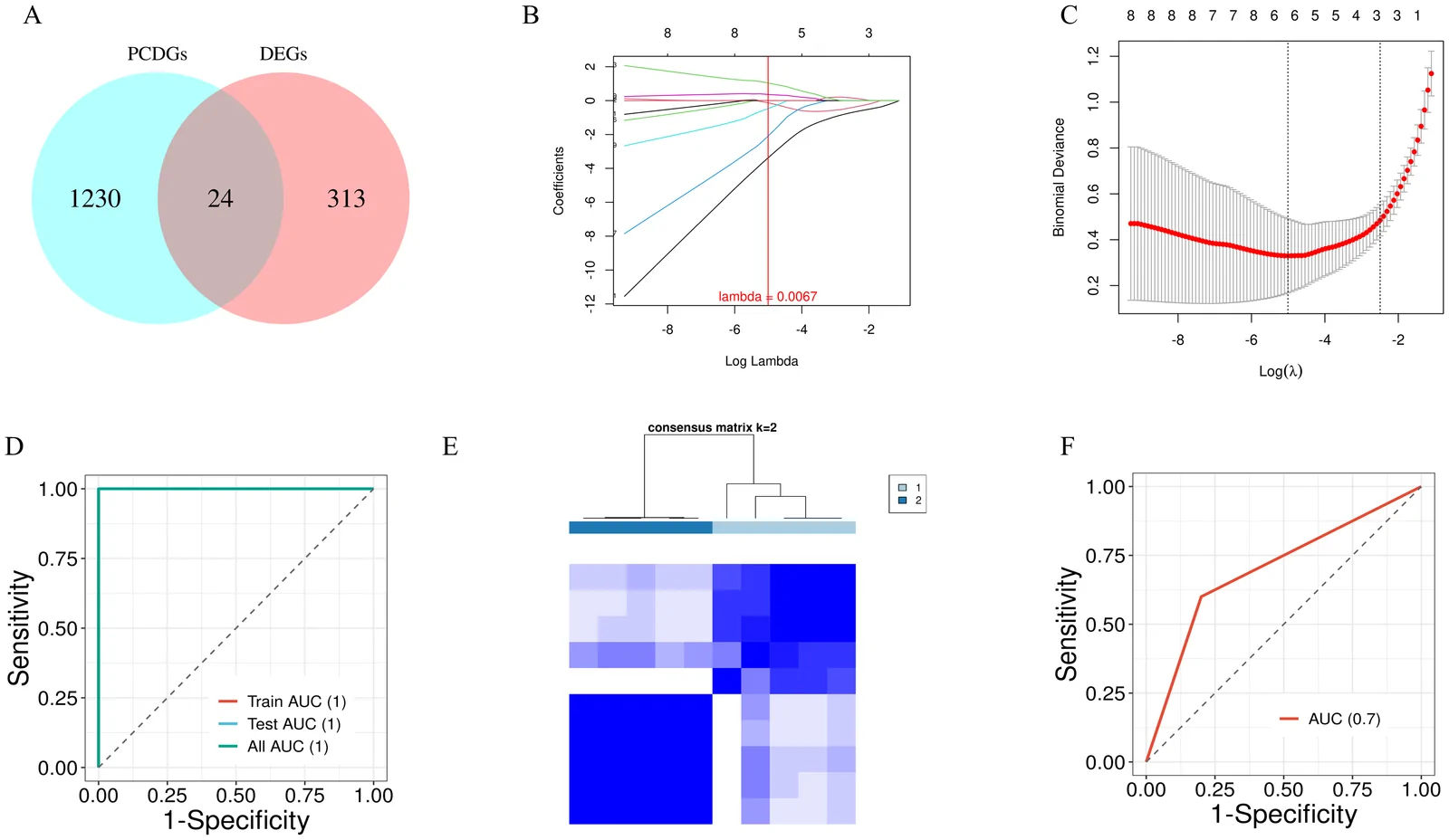
OA disease typing classifier training and external validation. **(A)** Venn diagram of OA differentially expressed genes intersected with PCD genes. **(B–D)** Construction of classifiers for disease subtypes by LASSO regression analysis. **(E, F)** External data validation based on the classification model obtained above.

Twenty-four PCD-related DEGs were identified by comparing the gene expression profiles between the two PCD molecular subtypes identified among OA samples (**Figure 5A**). We developed classifiers that can be used to distinguish different disease subtypes (**Figures 5B, C**) and validated them with ROC curves (Train AUC = 1, Test AUC = 1, All AUC = 1, bootstrap 95% CI: 1.00–1.00) (**Figure 5D**). The results indicated that the LASSO-based classifier showed strong discrimination capability for subtype classification. The calibration data were summarized and provided in **Supplementary Table 2**. Subsequently, we further validated the accuracy of the classifier by utilizing an external dataset GSE55235 for validation, clustering the external dataset (**Figure 5E**), and subsequently validating the ROC curve of the classifier (**Figure 5F**) (AUC = 0.7), further illustrating the potential use and accuracy of this classifier. This reduction may reflect differences in sample characteristics, biological heterogeneity, and technical platforms between cohorts.

### 3.7 Core gene screening associated with PCD

**Figure 6:**
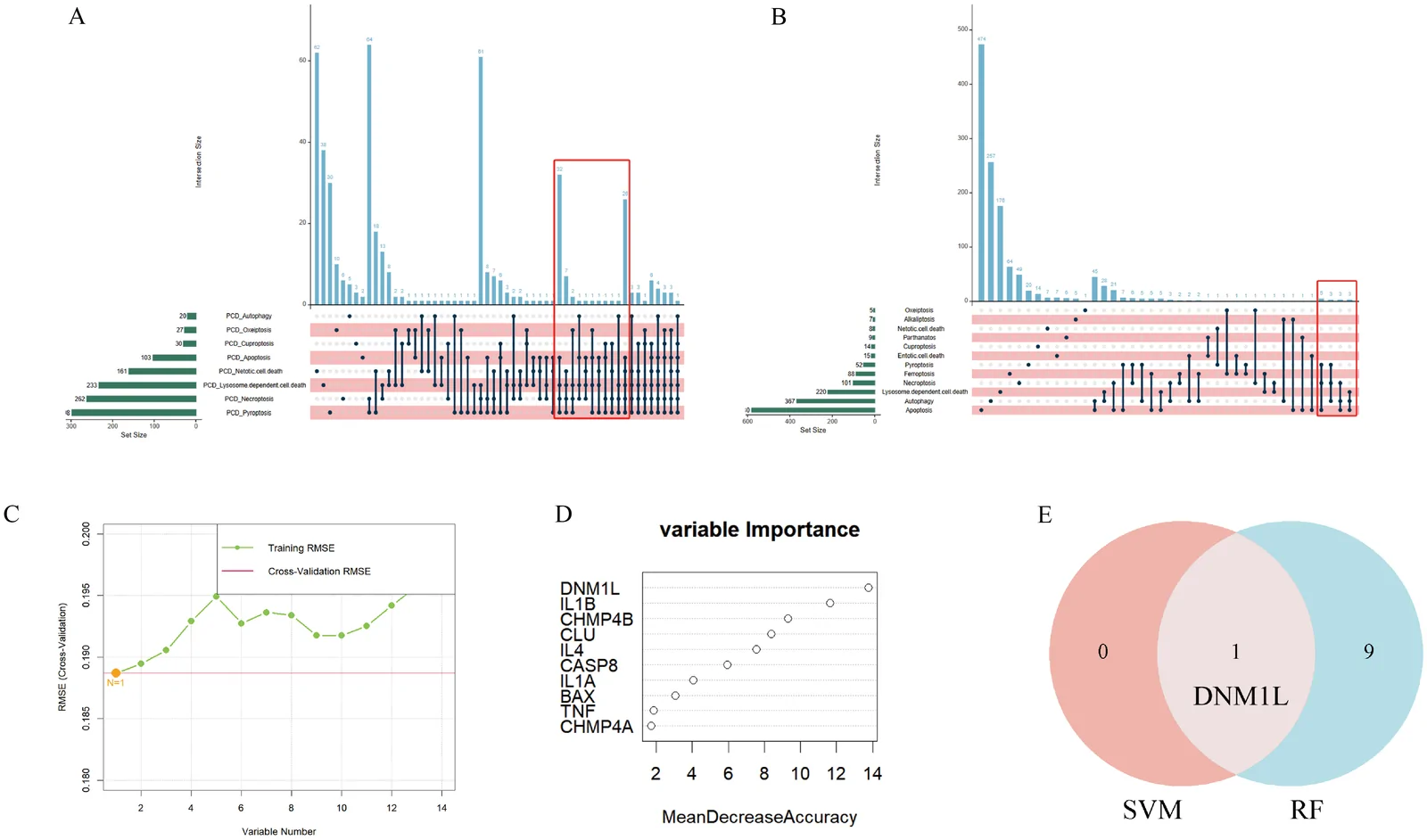
OA and PCD related core gene screen. **(A)** Intergroup difference analysis based on ssGSEA scoring of the intersection results of differential genes. **(B)** Intersection results of differential genes, plotted against the set of genes corresponding to the 12 PCD modalities taken for intersection. (Duplicates are mainly concentrated on the paths selected in the red box.) **(C)** A core gene screened using SVM. **(D)** A core gene ranked in the top 10 by applying RF screening. **(E)** Venn plots of the intersection of genes screened by SVM and RF.

To investigate differences in PCD activity between OA and control samples, ssGSEA scores for the 12 PCD modalities were compared between the two groups. Eight PCD modalities showed significant differences between OA and control samples (**Figure 6A**). For each significantly altered PCD modality, samples were further divided into high- and low-activity groups according to the median ssGSEA score, followed by differential expression analysis between the two groups. The resulting differential gene sets were compared using an UpSet plot to identify genes shared across multiple PCD modalities. The shared genes were mainly associated with apoptosis, netotic cell death, lysosome-dependent cell death, necroptosis, and pyroptosis, as highlighted by the red boxes (**Figure 6A**). These genes with differences were taken in intersection with the set of genes corresponding to the 12 PCD modalities, which showed that the number of duplicated genes in the 12 modalities was not too high, with Autophagy and Apoptosis and Lysosome-dependent cell death exclusive genes being more numerous (**Figure 6B**). These duplicate genes were mainly BAX, CASP8, IL1A, IL1B, TNF, CHMP4A, CHMP4B, HMGB1, BCL2, DNM1L, IFNG, CLU, IL4, LRRK2. Specifically, the 14 overlapping genes were derived from differential gene analyses associated with the 12 PCD modalities and used for OA-related hub gene screening, whereas the 24 PCD-related DEGs were identified between the two PCD molecular subtypes among OA samples and used for LASSO-based subtype classification.

### 3.8 Hub gene identification

**Figure 7:**
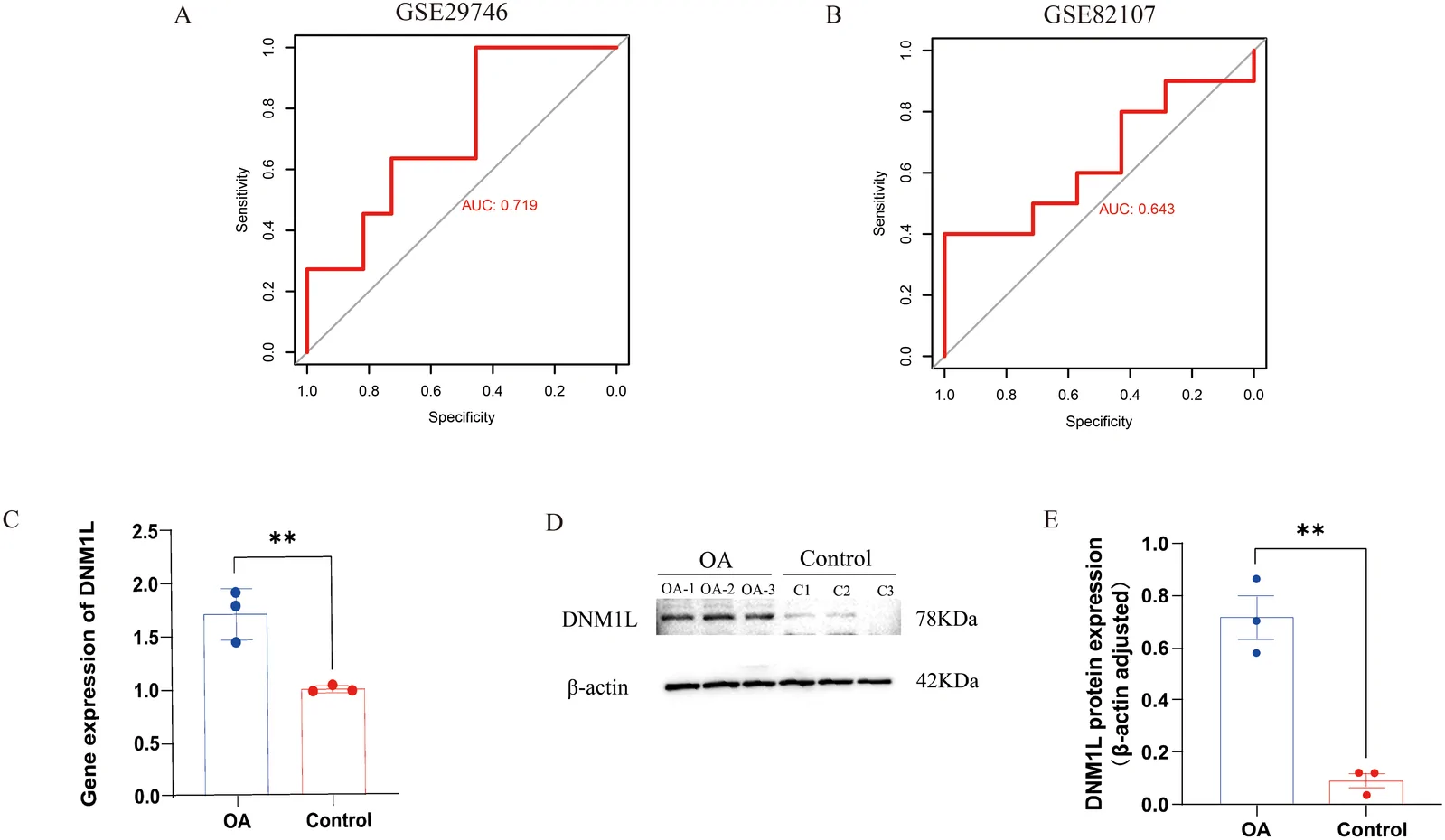
Identification and validation of hub gene. **(A, B)** Evaluation of the discriminative performance of core gene DNM1L across different datasets. **(C)** qRT-PCR results of DNM1L in OA patients versus controls. **(D, E)** Plot of WB results of DNM1L in OA patients and controls. ** Means P<0.01.

In order to further screen out PCD and OA-related HUB gene targets with significant eigenvalues, 2 different algorithms (SVM-RFE and Random Forest) were applied based on the above 14 genes. By SVM and RF, we finally obtained 1 HUB gene: DNM1L (**Figures 6C, D**). In the GSE29746 and GSE82107 datasets, DNM1L showed AUC values of 0.719 and 0.643, respectively, indicating a certain degree of discriminatory capability between osteoarthritis and control samples across independent cohorts (**Figures 7A, B**). However, the relatively modest AUC values suggest that DNM1L alone may not be sufficient as a standalone diagnostic biomarker, and further validation in larger clinical cohorts is required. The mRNA expression of DNM1L in synovial tissue was evaluated using qRT-PCR to compare gene expression between the control and osteoarthritis groups. Results demonstrated significantly higher DNM1L expression in the osteoarthritis group compared to the control group (**Figure 7C**). Immunoblot analysis confirmed these findings, revealing increased DNM1L protein expression in the osteoarthritis group, consistent with the qRT-PCR results (**Figures 7D, E**). The detailed qRT-PCR results are presented in **Table 1**.

**Table 1:** qRT-PCR experimental results.

Sample	Group	Ct Rep1	Ct Rep2	Ct Rep3	Mean Ct	SD	Intra-assay CV%​
C1	Control	36.9771957397461	35.3393669128418	35.4431953430176	35.9199180603027	0.917	2.55%​
C2	Control	28.1117649078369	27.9628067016602	28.115327835083	28.06329981486	0.087	0.31%​
C3	Control	27.6330680847168	27.6398811340332	27.5595111846924	27.610818862915	0.045	0.16%​
OA1	OA	28.0161190032959	27.3393096923828	27.606653213501	27.6540273030599	0.340	1.23%​
OA2	OA	32.3858451843262	32.6545219421387	32.8962135314941	32.6455268859863	0.255	0.78%​
OA3	OA	31.6819801330566	31.2747077941895	31.374963760376	31.443883895874	0.212	0.67%

## 4 Discussion

OA is a chronic condition (41) characterized by the progressive deterioration of articular cartilage and subsequent inflammation of the synovial cavity (42). The primary symptom associated with OA is pain; during the initial stages of this disease, pain is predominantly experienced during movement, while in the advanced stages, it manifests as persistent and compressive pain (43). Furthermore, osteoarthritis leads to joint stiffness and a reduction in motor function (42). Apoptosis has been observed to occur in the articular cartilage of many patients suffering from OA (44). Cell death is a complex process that includes various types of programmed cell death (PCD), such as Apoptosis, Autophagy, Cuproptosis, Entotic cell death, Ferroptosis, Lysosome-dependent cell death, Necroptosis, Netotic cell death, Oxeiptosis, Parthanatos, Alkaliptosis, and Pyroptosis. Studies have shown that chondrocyte apoptosis and inflammatory response are important factors in the pathogenesis of OA, and that the number of apoptotic chondrocytes correlates positively with the degree of OA-mediated degradation of human cartilage (31). Collectively, our transcriptomic correlation analysis suggests that dysregulated PCD is closely associated with chondrocyte loss and OA progression, implying that targeted PCD modulation may provide a potential theoretical direction for exploring OA pathological mechanisms. We therefore performed systematic bioinformatic analyses to screen candidate biomarkers and molecular loci with potential research value for OA pathological exploration.

We first identified two subtypes, Cluster 1 and Cluster 2, among OA patients based on the expression of individual genes associated with programmed cell death (PCD) and the corresponding gene sets used as references for OA samples. In our analysis of differentially expressed genes (DEGs) within the two subtypes, we observed an enrichment in pathways associated with viral myocarditis, cell adhesion molecules, protein digestion and absorption, and tuberculosis. The immune infiltration analysis revealed a greater degree of immune cell infiltration from memory B cells, M1 macrophages, and neutrophils in Cluster 1 patients, while Cluster 2 patients showed infiltration primarily from M0 macrophages, naïve B cells, and regulatory T cells (Tregs), with other cells exhibiting more significant correlations in infiltration levels. Subsequently, we conducted immune score, which indicated that the immuno-scores for Cluster 1 were significantly higher than those for Cluster 2. We analyzed the differences in immune checkpoints across the disease subtypes, revealing significant variations among a total of nine immune checkpoints, including HAVCR2, LAIR1, CD200R1, TIGIT, CD86, CD80, PVR, BTLA, and CD276. Subsequently, an IC50 drug analysis was performed for the various typologies, indicating that LY2109761, I-BET-762, Fludarabine and PFI3 exhibited differential predicted drug sensitivity across two PCD-related OA subtypes, which provides preliminary computational clues for subsequent compound-related OA research. To verify the stability of our PCD-based subtyping system, we constructed a classification model and validated its discrimination performance using an independent external transcriptomic dataset. Following this, we screened for core genes associated with OA and PCD, focusing on cell death modalities such as Apoptosis, Netotic cell death, Lysosome-dependent cell death, Necroptosis, and Pyroptosis. The primary overlapping genes included BAX, CASP8, IL1A, IL1B, TNF, CHMP4A, CHMP4B, HMGB1, BCL2, DNM1L, IFNG, CLU, IL4, and LRRK2. Subsequently, we utilized two machine learning methodologies, SVM-RFE and Random Forest (RF), to select features from these 14 genes, ultimately identifying DNM1L as a hub gene. To validate these findings, synovial tissue samples were collected from patients undergoing arthroscopic surgery, which were then categorized into control and osteoarthritis groups. Total RNA and protein were extracted from the synovial tissue, followed by quantitative real-time PCR (qRT-PCR) and Western blot analyses. The results indicated a significantly higher expression level of DNM1L in the osteoarthritis group relative to the control group, corroborating our earlier findings.

DNM1L (Dynamin-1-like protein), also referred to as DLP1 or DRP1, is a member of the dynamin GTPase superfamily (45) and serves as a critical regulator of mitochondrial fission (46). Mitochondrial dynamics, including both fusion and fission processes, are crucial for various mitochondrial functions, such as apoptosis, cellular stress responses, and autophagy (46). DNM1L is highly conserved and expressed in the cytoplasm of numerous cell types, where it is essential for mitochondrial fission. Upon activation, DNM1L translocates to the outer mitochondrial membrane and binds to the mitochondrial junction protein FIS1, facilitating the mitochondrial fission process (47). It is well-established that mitochondrial dysfunction induces excessive production of reactive oxygen species (ROS), promoting autophagy and exacerbating synovial inflammation via the upregulation of pro-inflammatory cytokines through NF-κB signaling activation (48). Research has demonstrated that DNM1L deficiency attenuates IL-1β-induced phosphorylation of AKT and IKK, as well as the nuclear translocation of NF-κBp65 (49). Therefore, it is reasonable to conclude that effective inhibition of DNM1L could represent a promising therapeutic approach for treating OA. Previous studies have demonstrated the importance of mitochondrial dysfunction in the pathogenesis of OA (50, 51), but the specific mechanisms need to be further clarified. One study reported increased expression and activation of DNM1L in cartilages of both human and mouse OA models, as well as in chondrocytes and cartilage explants treated with IL-1β, which correlated with excessive fragmentation of the mitochondrial network, oxidative stress, apoptosis, and degradation of the cartilage extracellular matrix (ECM). Importantly, the inhibition of DNM1L-mediated mitochondrial fission using mitochondrial division inhibitor-1 (Mdivi-1) effectively suppressed IL-1β-induced mitochondrial damage, cytochrome c release, and chondrocyte apoptosis *in vitro* (30). These findings strongly suggest that DNM1L plays a pivotal role in programmed cell death throughout the disease progression of OA. In this study, PCD activity was markedly elevated in osteoarthritic cartilage compared with healthy controls. Integrated bioinformatics and machine learning analyses identified DNM1L as a key PCD-associated gene, and its upregulation was confirmed at both the mRNA and protein levels by qRT-PCR and Western blotting. Given the established role of DNM1L (also known as DRP1) in regulating mitochondrial dynamics and multiple PCD modalities—including apoptosis, autophagy, pyroptosis, and ferroptosis—these findings suggest that aberrant DNM1L expression may reflect dysregulation of PCD-related pathways during OA progression. Nevertheless, the present study reveals only a correlative relationship between DNM1L dysregulation and PCD phenotypes; definitive causal mechanisms remain to be established. This association is supported by previous work using the destabilization of the medial meniscus (DMM) mouse model, in which increased DRP1/DNM1L expression coincided with excessive mitochondrial fission, chondrocyte apoptosis, and extracellular matrix degradation, thereby implicating abnormal mitochondrial fission in OA pathogenesis (30, 52). Despite these insights, therapeutic targeting of DNM1L/DRP1 remains underdeveloped. The only published evaluation of the mitochondrial fission inhibitor Mdivi-1 in the DMM model employed systemic intraperitoneal administration and focused exclusively on cartilage integrity, without assessing synovial inflammation or OA-related pain behaviors. Furthermore, the efficacy of Mdivi-1 has not been examined in the monosodium iodoacetate-induced OA model (30, 52, 53). Accordingly, functional evidence for mitochondrial fission inhibition in preclinical OA remains fragmented, with a lack of comprehensive, multi-dimensional *in vivo* assessments that integrate structural, inflammatory, and pain-related endpoints. Collectively, our data—together with existing literature—support a close link between DNM1L dysregulation and PCD disturbance in OA chondrocytes. Targeted inhibition of DNM1L/DRP1 represents a plausible disease-modifying strategy that may concurrently attenuate cartilage degeneration, synovitis, and pain, pending rigorous validation in physiologically relevant *in vivo* models.

Although our transcriptome analysis combined with clinical synovial tissue validation provides correlative evidence linking PCD and DNM1L to OA pathogenesis, the present study still has several inherent limitations that should be objectively acknowledged. First, although independent cohorts were used for validation, the discovery cohort contained relatively fewer healthy controls, which may introduce potential sampling bias. Two PCD-related molecular subtypes were defined by consensus clustering based on PCD-related ssGSEA profiles. Subsequently, the LASSO classifier was constructed using PCD-related genes differentially expressed between these same subtypes and evaluated in the same cohort. The perfect discrimination observed in the internal cohort (AUC = 1.000) is the result of internal/circular validation. In the external GSE55235 cohort, the AUC decreased to 0.700, indicating limited generalizability across cohorts. Thus, the LASSO-based classifier should currently be considered an exploratory and hypothesis-generating tool rather than a clinically validated subtype classification model. Furthermore, the clinical validation cohort consisted of only three OA patients and three control individuals, which limits statistical power. Therefore, these findings should be regarded as preliminary validation of DNM1L expression. Larger age-matched clinical cohorts, together with functional studies using OA-relevant cellular models and animal models, are needed to further clarify the biological and clinical significance of DNM1L in OA. Second, the GDSC2 database was constructed from *in vitro* cancer cell lines rather than primary OA tissues. Therefore, the subtype-associated compounds identified in this study represent candidate agents for OA treatment that warrant future experimental validation. Third, the biological functions and molecular mechanisms of DNM1L in osteoarthritis require further investigation through *in vitro* and *in vivo* experiments.

## 5 Conclusion

PCD is increasingly implicated in the pathological progression of OA. Correlative multi-omics analyses from the present study reveal that aberrant PCD signatures are robustly associated with chondrocyte damage and OA degenerative lesions, which implies that targeted PCD regulation could represent a promising theoretical research avenue for subsequent mechanistic dissection and therapeutic development of OA. Herein, we stratified OA patients into two distinct PCD-relevant molecular subtypes (Cluster 1 and Cluster 2) and further identified and experimentally verified DNM1L as a core hub gene tightly linked to OA pathogenesis. The PCD-based subtyping system and DNM1L candidate biomarker established in our research deliver correlative molecular evidence to facilitate follow-up mechanistic studies and drug discovery efforts against OA. Accordingly, DNM1L expression may offer preliminary pathological insights and serve as a candidate biomarker for OA, pending validation in larger independent cohorts.

## Data Availability

The datasets presented in this study can be found in online repositories. The names of the repository/repositories and accession number(s) can be found in the article/**Supplementary Material**.
